# Increased corticospinal excitability after rest with short term motor performance

**DOI:** 10.1113/EP093874

**Published:** 2026-09-18

**Authors:** Quinn McCallion, Alistair R. Clark, Bryan S. Barry, Emelia E. Duchow, Brach Poston, Zachary A. Riley

**Affiliations:** ^1^ School of Health and Human Sciences Indiana University‐Indianapolis Indianapolis Indiana USA; ^2^ Department of Kinesiology and Nutrition Sciences University of Nevada‐Las Vegas Las Vegas Nevada USA

**Keywords:** central fatigue, corticospinal excitability, learning, plasticity

## Abstract

Motor skill performance is associated with early changes in corticospinal excitability (CSE), yet the influence of rest periods surrounding motor activity on these neurophysiological responses remains unclear. This study examined how matched pre‐ and post‐activity rest intervals influence CSE following novel fine motor task performance. Twenty‐one healthy adults (18–39 years) participated in a single‐session, within‐subject protocol consisting of a 30‐min rest period (R1), 30 min of videogame‐based motor activity using a guitar controller (A1), and a second 30‐min rest period (R2). Transcranial magnetic stimulation combined with surface electromyography assessed corticospinal excitability and activity of intracortical circuits in the first dorsal interosseous (FDI) and abductor digiti minimi (ADM). Gameplay performance significantly improved during the activity block, demonstrating short‐term performance improvements took place. The 1mVSTIM MEP measure in FDI demonstrated a significant main effect across the three time points in the block, and pairwise comparisons showed that the 1mVSTIM MEP was significantly larger after R2 when compared with R1, and for R2 compared to A1. There were no significant changes in long interval intra‐cortical inhibition, short intracortical inhibition or intracortical facilitation for the FDI. No changes were detected in the ADM. These findings suggest that adequate pre‐ and post‐task rest may facilitate increased corticospinal excitability following novel motor task performance without altering measures of inhibition or facilitation.

## Abstract (highlights)

- **What is the central question of this study?**
  How do rest periods before and after short term motor performance affect changes in corticospinal excitability and intracortical circuits?
- **What is the main finding and its importance?**
  Adequate rest after short term motor performance may facilitate the early learning processes that stabilize motor memories. This could play an important role in normal skill acquisition and adaptations with rehabilitation.

## 1 INTRODUCTION

Motor skill acquisition is traditionally understood as a process whereby sustained and repeated practice leads to gradual improvements in performance, and is accompanied by neural adaptations within the motor system (Luft & Buitrago, 2005; Newell, 1991). Emerging evidence suggests that the early stages of novel task performance are also accompanied by rapid and transient changes in cortical and corticospinal excitability (CSE), particularly within the primary motor cortex (M1) (Dayan & Cohen, 2011; Kantak et al., 2013; Manto et al., 2006; Winkler et al., 2012). Short‐term motor performance represents the initial phase of skill acquisition and can be observed and quantified during a single session of motor practice (Soderstrom & Bjork, 2015). Excitability refers to the responsiveness and functional readiness of the nervous system to produce a motor command (Weavil & Amann, 2018). A common method for assessing the neurophysiological changes in CSE is through the generation of a motor evoked potential (MEP). MEPs are electromyographic (EMG) responses recorded from peripheral muscles following the application of transcranial magnetic stimulation (TMS) over the representation of the target muscle within the contralateral hemisphere of M1 (Spampinato et al., 2023). The characteristics of the TMS‐evoked responses following motor activity depend on the stimulation paradigm employed. While single‐pulse TMS provides an assessment of overall CSE, paired pulse conditioning paradigms, utilizing a subthreshold conditioning stimulus delivered prior to a supra‐threshold test stimulus, provide insight into the excitatory and inhibitory state of the nervous system in response to motor activity (Ridding et al., 1995).

Measuring the overall amplitude of an MEP can aid in the characterization of the early CSE associated with novel, short‐term motor skill performance. The current literature has shown increases in MEP amplitude following the practice of fine motor isometric target reaching or tracking tasks utilizing pinch grip, index finger abduction, or wrist flexion and extension (Cirillo et al., 2020; Coxon et al., 2014; Fujiyama et al., 2017; Mooney et al., 2019), whereas others such as Paparella et al. (2020) and Smyth et al. (2010) reported no change in CSE with the similar motor activities. These inconsistencies in the literature indicate the need for further exploration of CSE measures in relation to fine motor performance. Intracortical facilitation (ICF) is a neurophysiological process whereby the excitation of neural pathways within the primary motor cortex can enhance the transmission of centrally derived movement signals to the alpha motor neurons for transport to the periphery (Chen et al., 1998). ICF and MEP amplitude can change following motor performance, but there can be a dissociation between them. This may be due to the potential differences in sensitivity between ICF and MEP to different task characteristics, that is, skilled versus non‐skilled (Ho et al., 2022). During the preparation and execution of motor tasks, paired pulse TMS can be used to assess the state of corticocortical pyramidal cells and their excitatory glutamatergic synapses as they potentially alter intracortical excitability through facilitating the activities of other neurons (Keller, 1993). Recently, Ho et al. (2022) and Mooney et al. (2021) observed decreased ICF after a long session of motor skill learning, with unaffected measures of MEPs and proposed that a diminished ICF was recorded in response to the beginnings of the stabilization process for the motor memory of the task. This stabilization process is thought to be initiated within an hour of task performance and highlights the importance of rest following repetitive task execution (Xu et al., 2009). The difference in MEP and ICF amplitude demonstrates the interdependent relationship between ICF and MEP and exemplifies how changes in ICF may not give a complete explanation of the overall responsiveness of the entire motor pathway during motor task performance (Malcolm et al., 2015).

Similarly, a change in intracortical inhibition (ICI) can be observed independent of a change in MEP amplitude following motor skill performance (Hamel et al., 2023). ICI represents the simultaneous decreased firing of neighboring neurons within the cerebral cortex (Leodori et al., 2019). This can allow the suppression of unwanted neural activity and in turn unwanted muscle action, resulting in the ability to more readily execute precise fine motor tasks with greater proficiency (Ding et al., 2018). Using different paired pulse TMS paradigms, ICI can be recorded as short (SICI) and long (LICI), assigned based on the length of the interstimulus interval (ISI) (Premoli et al., 2018). While both SICI and LICI are due to reduced cortical excitability, they are thought to be two distinct circuits that mediate the activity of different cortical cell populations (Sanger et al., 2001). Through the activity of pre‐ and postsynaptic GABAergic B receptors (activated during LICI) or postsynaptic GABAergic A receptors (activated during SICI), it has been proposed that SICI and LICI may have different effects on early motor performance, cortical excitability, plasticity and overall output to the spinal cord and the periphery (Chen et al., 1998; Mott & Lewis, 1994; Sanger et al., 2001; Ziemann et al., 1998). Reduced expression of GABAergic influences has previously been associated with short term motor performance (Jacobs & Donoghue, 1991). The disinhibition of SICI during a motor task has been proposed to enhance long term potentiation (LTP)‐like processes and give rise to greater CSE (Barron, 2021). However, as with other cortical measures, different authors have also reported findings with no significant changes or increases in ICI with early motor skill performance, suggesting that ICI may be an influential but not definitive factor in observing greater CSE (Cirillo et al., 2010, 2020; Rogasch et al., 2009).

Discrepancies in the findings between measures of CSE and short‐term motor skill performance may be related to the subtle differences in methodology, including task paradigms, measurement intervals and rest parameters. Little research has been performed investigating the short‐term performance of a repetitive fine motor task and the accompanying cortical changes when the experimental procedure includes a time‐matched period of rest pre‐ and post‐activity. In a study performed by Avanzino et al. (2011), a demanding finger tapping task was used to observe how changes in motor performance may correlate with CSE. Initially participants were divided into three groups. All of the groups had their baseline MEP amplitudes measured without an initial rest period, and two of the groups had their follow up MEPs measured after 5 min of a fine motor, demanding task. Following this measurement one of the groups performed a second block of the finger tapping task for 1 min, and the other group performed the finger tapping task for another 5 min. Researchers observed a deterioration of MEP amplitude following the initial 5‐min demanding task, which was not able to recover during a 5‐min rest period, even though motor performance was able to. However, after 15–20 min of rest, CSE was able to recover back to baseline levels, thereby suggesting there is a dynamic interaction between measures of CSE, short‐term motor performance and rest. Extension of the rest periods surrounding the motor activity, although not all explicitly measured in the study, may have served to mitigate fatigue‐related suppression of the nervous system, facilitate recovery of excitability and support improvements in motor task.

While the study conducted by Avanzino et al. (2011) highlights the important, and potentially multifaceted role that rest may play in the measured levels of CSE following a motor task, the typical cognitive load of activities of daily living (ADL) can change resting CSE (Buss et al., 2023). Because resting CSE is measured prior to the onset of a motor task, differences in type and volume of ADL can cause a misrepresentation of resting CSE, in turn affecting the change in CSE observed with activity. Incorporating an initial period of rest prior to activity may, therefore, provide a more representative baseline measure of CSE, allowing for a more accurate assessment of task‐induced changes to CSE. The results from Avanzino et al. (2011) agree that the length of rest following the performance of a task is important for CSE recovery, but the discussion does not consider the potential impact an initial rest period may have on excitability changes. If a time‐matched post‐task rest is necessary for CSE to return to baseline following the motor activity, it is plausible that a time‐matched pre‐task rest may also serve a similar function. By allowing preceding elevations or suppressions in CSE from ADLs to be overcome before baseline measurements are obtained, a more accurate depiction of how CSE is changing with a chosen task may be achieved. Overall, this simply suggests that sufficient rest may be necessary to normalize excitability levels regardless of the preceding stimulus originating from a structured motor task or ADL. Furthermore, although not directly measured by Avanzino et al. (2011), this recovery of CSE may be important for initiating physiological processes such as disinhibition of intracortical GABAergic inhibitory circuits and increased LTP‐like processes involved with performance improvements (Barron, 2021; Cantarero et al., 2013; Versace et al., 2021). Therefore, further investigation into the influence of rest dynamics on CSE recovery is needed.

Premised with the rationale that rest can impact motor task performance and CSE differently, the present study aims to determine the effects of matched duration pre‐ and post‐activity rest periods on measures of CSE. We hypothesize that the introduction of time‐matched pre‐ and post‐activity rest will support an increase in CSE immediately after activity and after post‐activity rest. Clinically, patients with neurological disorders undergo the relearning of ADLs such as driving a car, shaving, or texting on a phone. The relearning of an ADL is maximized by performing the greatest number of repetitions possible during a therapy session (Zhan et al., 2018). This aids in consolidating the motor task that was performed and can improve functional outcomes (Yu et al., 2024). However, often within clinical settings this is achieved with little consideration of the relationship between short‐term motor performance and CSE. For example, it has been proposed that children born preterm are able to engage compensatory mechanisms, allowing them to achieve the same functional motor goal with diminished levels of CSE, and potentially without activating the same stable cortical pathways for potentiating long‐term learning and consolidation of the task (Pitcher et al., 2012). If this is also true for other clinical conditions or healthy individuals with fatigue‐induced decreases in CSE, it may reduce retention and motor performance in follow‐up sessions, negatively impacting patient progress in therapy. However, if layering rest periods around intervals of motor activity can negate decreases in CSE, it may result in the maintenance of the physiological adaptations that accompany the short‐term performance of a motor task. Therefore, this study will investigate the effects of introducing rest periods before and after the short‐term performance of a motor task on CSE.

## 2 METHODS

### 2.1 Participants

Twenty‐one healthy adults (males: *n* = 6 and females: *n* = 15), ages 18–39 with no history of significant neurological disorder, skeletal muscle disorder or stimulant consumption at least 12 h prior to their visit were recruited for this study. On the first lab visit, subjects completed a brain stimulation questionnaire to determine eligibility to participate (Rossi et al., 2021, 2009). Subjects were then asked to read the informed consent, and researchers answered any questions prior to the subject signing. The study was approved by the Indiana University Institutional Review Board (approval reference no. 27959) and was conducted according to the *Declaration of Helsinki*, except for registration in a database. After completing these steps, subjects filled out a gaming experience questionnaire and an Edinburgh Handedness Inventory Assessment to determine hand dominance before beginning the task. All subjects were right‐handed guitar players (*R* > 40) (Oldfield, 1971). The weekly gaming experience for the 21 subjects tested was: 0 h (*n* = 5), 0–1 h (*n* = 8), 1–3 h (*n* = 5), 3–6 h (*n* = 2) and 6–10 h (*n* = 2).

**FIGURE 1:**
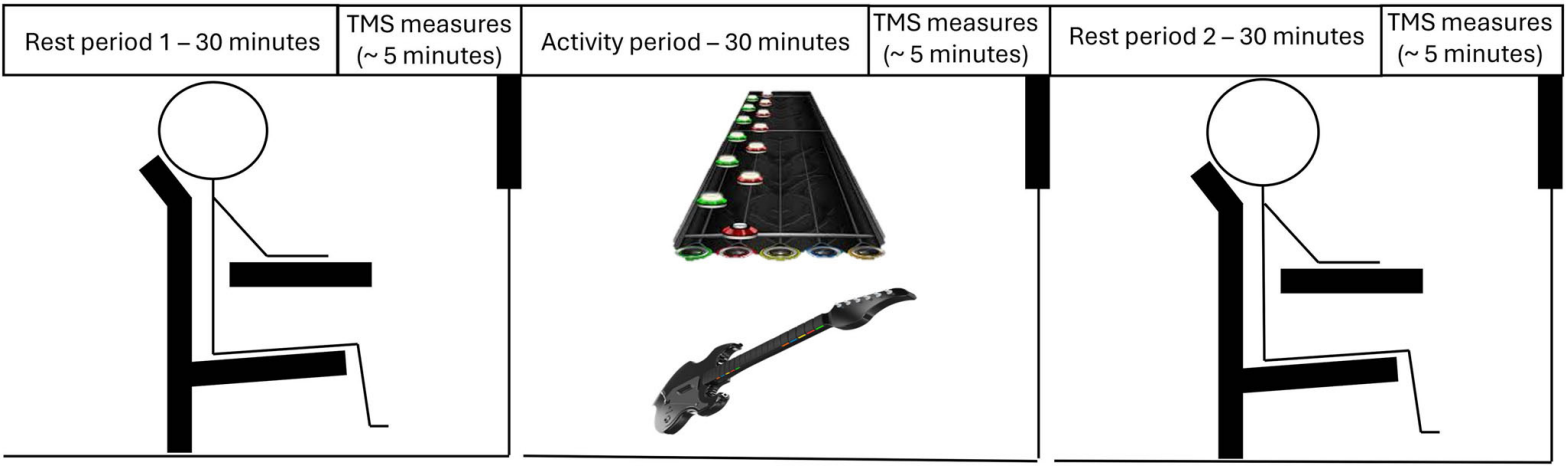
Timeline and visual of the videogame‐play.

The study utilized a within‐subject design where participants represent their own control. All subjects participated in a single session that had three trial blocks. The three trial blocks consisted of an initial rest block of 30 min (rest block one; R1), an activity block where they played a videogame for 30 min (activity block 1; A1), and a second rest block of 30 min (rest block 2, R2; see Figure 1). After each block, participants had four different measures taken with TMS before moving on to the next block.

### 2.2 Electromyography

Surface electromyography (sEMG) was applied using anatomical landmarks and palpation to the first dorsal interosseus (FDI) and abductor digiti minimi (ADM) muscles. Bipolar electrodes were secured on the muscle bellies. These muscles were recorded as they assist in the movement of the index and pinky fingers for the videogame task in the second block of activity. To ensure signal clarity a reference electrode was placed over the styloid process of the ulna of the same wrist. A Delsys (Boston, MA, USA) EMG unit connected to a Cambridge Electronic Design (CED; Cambridge, UK) Micro 1401‐4 data acquisition unit was used to collect and convert analog EMG data at a rate of 1000 Hz to digital and record in Spike2 software (CED) for processing. That data were then exported for further analysis in MATLAB (MathWorks, Natick, MA, USA).

### 2.3 Transcranial magnetic stimulation

A Magstim (Whitland, UK) BiStim 2 transcranial magnetic stimulator with a figure‐of‐eight coil was used in conjunction with EMG to assess the excitability of the cortical pathways between the motor cortices and FDI and ADM muscles of the hand. Since positioning the coil tangentially on the scalp with the handle pointing posteriorly and angled at approximately 45° to the midline has been previously shown to be the most effective at eliciting consistent TMS evoked potentials (TMS‐MEP) from the hand regions of the motor cortex (Deng et al., 2013; Gomez‐Tames et al., 2018), all stimulations were performed at this orientation. At the beginning of each session, a soft, flexible tape measure was used to measure subjects’ heads from tragus to tragus and nasion to inion to find and mark the skin above the vertex of the subject's skull. From the vertex, the motor cortex regions were then approximated by measuring ∼5 cm lateral and ∼1 cm rostral in accordance with previously established methods (Beyh et al., 2024). Single‐pulse stimuli at ∼40–60% of stimulator intensity were delivered within this vicinity until a location producing a consistently robust EMG response at a fixed stimulator intensity was observed.

### 2.4 Resting motor threshold and TMS evoked potentials

Following placement of the sEMG, participants had the non‐dominant hand region of M1 marked (C3 and C4 according to 10–20 EEG Coordinate System). Single pulse, biphasic TMS stimuli at ∼50–55% of simulator intensity was delivered using a hand‐held figure‐of‐eight TMS coil powered by a Magstim BiStim^2^, to confirm the location of the somatotopic areas of the cortex associated with FDI. This process was performed on the contralateral hemisphere (right) of the non‐dominant hand (left) of each subject, perpendicular with the central sulcus at an angle of 45° to induce a posterior to anterior current. Once located, each site was dotted with a second mark (if needed) for easy tracking. The resting motor threshold (RMT) was determined by applying single pulse TMS to the recently marked M1 site using a stimulation intensity of 30% of stimulator output. Stimulator intensity was progressively increased by increments of 5% of maximal stimulator output (30%, 35%, 40%, etc.) until a TMS‐MEP was observed in the corresponding FDI EMG. After this, stimulator intensity was reduced by 5% of maximal stimulator output and then slowly increased by increments of 1% of maximal stimulator output until an intensity was established that evoked a significant TMS‐MEP on ∼5 out of 10 stimulations (Torii et al., 2019). Beginning at 55% of stimulator output and adjusting by increments of 1%, the percentage of maximal stimulator output needed to elicit a 1 mV peak to peak (1mVSTIM MEP) was then found in each subject. The intensity for the 1mVSTIM MEP response has been used across many of our previous studies and has proved to be highly reliable (de Albuquerque et al., 2024; Greenwell et al., 2025; Wilkins et al., 2025, 2024).

During each of the cortical excitability testing blocks, 60 stimuli were triggered and delivered by the researchers at a frequency of greater than 1 Hz, depending on the stimulator output. Subjects were tested for measures of cortical inhibition and facilitation by the application of four diagnostic measures to the cortical region of the FDI. Testing measures were visually inspected after application of each stimulus and included 15 single‐pulse stimuli at 1mVSTIM MEP intensity and 15 paired pulse stimuli delivered to test short interval intracortical inhibition (SICI), 15 paired pulse stimuli delivered to test long interval intracortical inhibition (LICI) and 15 paired pulse stimuli delivered to test intracortical facilitation (ICF) for each subject (McCallion, 2026). Approximately 30–60 s was given between each testing block of 15 stimuli to adjust the stimulators and ensure the stimulation frequency was not inducing LTP or long‐term depression (LTD). For this reason, randomization or counterbalancing of the four different TMS tests was not considered warranted, and therefore TMS tests were performed in the same order for each participant. The SICI test involved a subthreshold conditioning stimulus (CS) at 90% RMT paired with a suprathreshold test stimulus (TS) at 1mVSTIM MEP intensity, and using an ISI of 3 ms (de Albuquerque et al., 2024). The LICI test employed a suprathreshold CS and TS each delivered at a 1mVSTIM MEP intensity with an ISI of 150 ms (Chu et al., 2008). Finally, much like the SICI test, the ICF will utilize a subthreshold CS at 90% RMT paired with a suprathreshold TS at 1mVSTIM MEP intensity. However, for this condition, an ISI of 10 ms was used (Pantovic et al., 2023). The four diagnostic conditions were administered at the end of each block (R1, A1, R2) during each subject's visit. Each time, measures of cortical excitability and inhibition were taken; data were recorded and averaged.

### 2.5 Rest

During the R1 and R2 blocks participants were sat in an ergonomically comfortable position where they could relax their non‐dominant hand on a side table. Subjects were encouraged to relax their arm and hand during the entire block and not to think about using that hand. To ensure distraction from using that hand, researchers played an ocean documentary. If the subject needed to use a hand (e.g., scratch, adjust clothing, etc.) they were instructed to use the other hand.

### 2.6 Clone Hero

During this study our focus was to generate a period of consistent activity while also being able to measure performance outcomes. To do this we used a bimanual coordination and timing task which allowed us to quantify the change in accuracy to a change in skill. Participants played Clone Hero, an open‐source alternative to Guitar Hero, on a computer using a guitar‐shaped controller. They positioned the index, middle, ring and pinky fingers of their non‐dominant hand on four coloured fret buttons, while the dominant hand rested on the strum button located on the body of the controller.

The game displayed four vertical lanes on the screen that corresponded in colour and order to the four fret buttons on the controller (see Figure 1). During gameplay, notes move downward along one or more of these lanes toward a target area at the bottom of the screen. To score points, participants pressed the matching fret button(s) with their non‐dominant hand and strummed with their dominant thumb. To achieve maximum points, the moving note had to be fully aligned with the target area.

Participants completed a 30 s familiarization period where the controls of the game were clearly explained and demonstrated. Participants then completed a 30 min activity period where songs lasting between 3.5 and 5.5 min were completed. Three different songs were used, and each song was completed once, before all songs were repeated for a second time. Following the completion of each song, the subject's performance data were recorded and they were provided a short rest period (∼30 s), to prevent musculoskeletal fatigue. The total number of ‘notes’ hit with each finger for the entire 30 min activity period were: index finger, 580; middle finger, 960; ring finger, 1140; pinky finger, 730. Data recorded from the game included accuracy (which is a measure of notes hit/total notes, total number of notes hit), best continuous streak, and overstrums (which records missed attempts to hit the note) (McCallion, 2026). From these metrics we were able to track the learning progression throughout the three songs, even though again, the main goal of using the motor task was to ensure the participants were consistently moving the digits of their non‐dominant hand and tracking performance was secondary.

### 2.7 Data analysis

The average peak‐to‐peak amplitude (mV) of the 15 stimulations for each TMS measure (1mVSTIM MEP, SICI, LICI, ICF) following the initial rest period, activity period and second rest period was calculated. Each of the paired‐pulse measures were normalized relative to the 1mVSTIM MEP measure during that same block and expressed as a percentage of 1mVSTIM MEP. The total number of hours per week subjects spent playing any type of video games was collected from the gaming experience questionnaire filled out at the beginning of the study. Accuracy, best continuous streak and the number of overstrums were recorded as the performance data during the activity portion of the study. Data from Spike2 were uploaded and processed with a custom MATLAB script. EMG data were sampled at a frequency of 1000 Hz. Within the custom script MEP onset was set to 0.015 s after the administration of the test pulse, window width was set to 0.2 s, the maximum and minimum values within that time window were extracted. This was repeated following each test pulse. The peak to peak was recorded as the difference between the maximum and minimum value within that window. Fifteen peak‐to‐peak measures were recorded for each test. The 15 peak‐to‐peak measures for each test were averaged individually (for the paired pulse tests, the 15 raw peak‐to‐peak values were normalized against the subject's unconditioned MEP in the same block before averaging). This resulted in four average peak‐to‐peak values for each testing block. This process took place through a combination of automatic processing and visual inspection.

### 2.8 Statistical analysis

First, for each muscle, a 3 × 4 repeated‐measures ANOVA was performed with testing block (R1, A1, R2) and TMS measure (1mVSTIM MEP, SICI, LICI, ICF) as within‐subject factors, to determine if there were significant interactions showing that the changes across the blocks depended on the TMS measure. Then, separate repeated‐measures ANOVAs were run on each TMS measure, as they had different scaling. To control for the familywise error rate of running four separate ANOVAs, we performed a Bonferroni correction to the main effect of block by manually dividing the α = 0.05 by 4 (different TMS measures) such that the new level of significance for this main effect was *P* < 0.0125. A Bonferroni correction was also applied to the pairwise comparisons if there was a significant main effect. Mauchly's test was used to assess sphericity, and Greenhouse–Geisser corrections were applied when this assumption was violated.

Changes in videogame performance were analysed with a paired Student's *t*‐test between the averages of the first three songs and the last three songs during the Clone Hero activity block. These comparisons were made for accuracy, best continuous streak, and overstrums. Finally, correlations were performed on changes in gameplay accuracy and changes in each TMS measure (R2–R1) to explore relationships between the variables. All analyses were performed in SPSS Statistics 29 (IBM Corp., Armonk, NY, USA) and a *P*‐value of 0.05 was considered significant, unless denoted with the manual Bonferroni correction.

## 3 RESULTS

### 3.1 FDI

**FIGURE 2:**
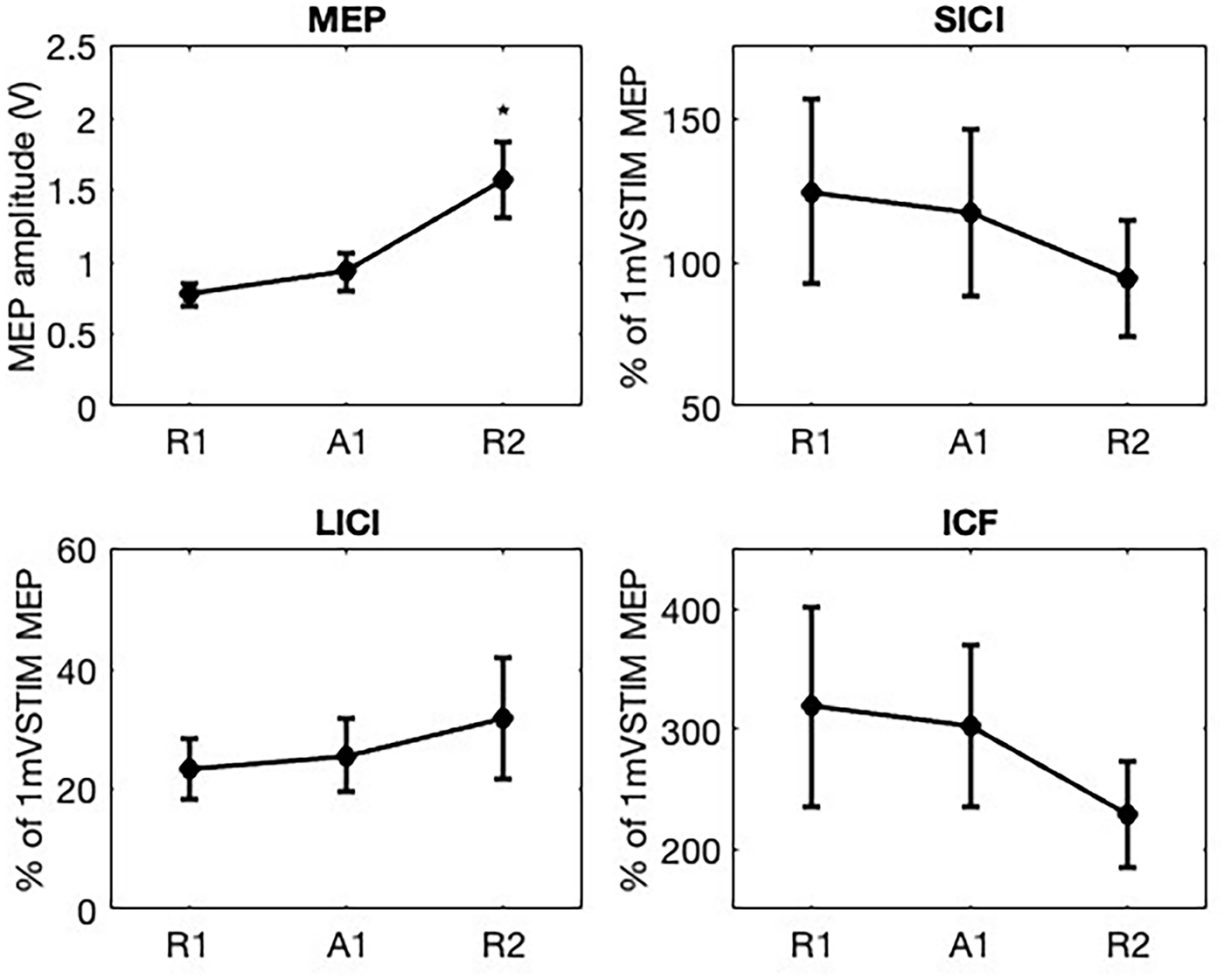
Results from the single‐pulse MEP (top‐left) and the paired‐pulse paradigms (SICI, LICI, ICF) from the FDI during the three blocks. *Significance of *P *< 0.05 between blocks R1–R2, and blocks R1–A1 in the MEP. Data represent the mean ± standard error.

For the FDI muscle, the 3 (testing block) × 4 (TMS measure) repeated measures ANOVAs showed there was no significant interaction between the testing block and TMS measure (*F*(6, 120) = 0.721, *P* = 0.538, η_p_^2^ = 0.025). The 1mVSTIM MEP measure in FDI, or what would be the standard single‐pulse MEP, demonstrated a significant main effect (*P* < 0.0125, Bonferroni corrected) across the three time points in the block (*F*(2, 40) = 6.826, *P* = 0.008, η_p_^2^ = 0.254; Figure 2). Pairwise comparisons showed that the 1mVSTIM MEP was significantly larger after R2 when compared with R1 (*P* = 0.005), and for R2 compared to A1 (*P* = 0.03). There were no main effects across blocks for SICI (*F*(2, 40) = 0.460, *P* = 0.554, η_p_^2^ = 0.022), LICI (*F*(2, 40) = 0.446, *P* = 0.569, η_p_^2^ = 0.022), or ICF (*F*(2, 40) = 0.789, *P* = 0.433, η_p_^2^ = 0.038).

### 3.2 ADM

**FIGURE 3:**
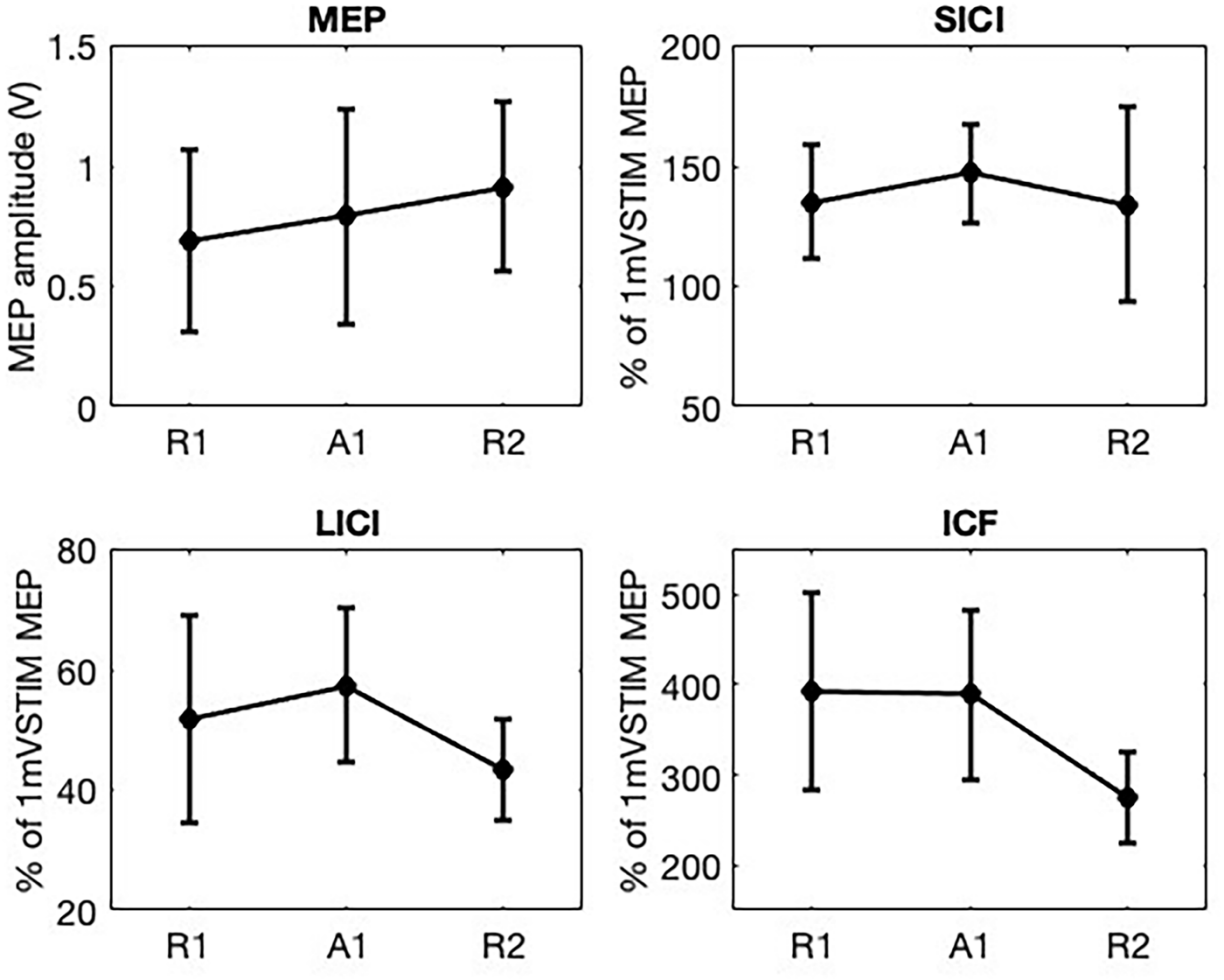
Results from the single‐pulse MEP (top left) and the paired‐pulse paradigms (SICI, LICI, ICF) from the ADM during the three blocks. There were no significant differences in any of the measures. Data represent the mean ± standard error.

For the ADM muscle, the 3 (testing block) × 4 (TMS measure) repeated measures ANOVAs also showed there was no significant interaction between the testing block and TMS measure (*F*(6, 120) = 0.638, *P* = 0.531, η_p_^2^ = 0.030). Contrary to the FDI, the 1mVSTIM MEP measure in ADM showed no significant main effect across the three time points in the block (*F*(2, 40) = 1.229, *P* = 0.290, η_p_^2^ = 0.058; Figure 3). There were no main effects across blocks for SICI (*F*(2, 40) = 0.132, *P* = 0.845, η_p_^2^ = 0.007), LICI (*F*(2, 40) = 0.574, *P* = 0.539, η_p_^2^ = 0.028) or ICF (*F*(2, 40) = 0.650, *P* = 0.506, η_p_^2^ = 0.031).

### 3.3 Gameplay

For A1, which was the Clone Hero videogame playing block, we measured three different variables averaged across the first three song trials and the last three song trials. Those results are shown in Table 1. Briefly, there were significant increases in the accuracy (percentage of correct notes played; *t*(20) = –6.540, *P* < 0.001) and best continuous streak of notes played (*t*(20) = –2.386, *P* = 0.013), while the number of overstrums decreased (*t*(20) = 1.982, *P* = 0.030). There were no significant correlations between changes in gameplay accuracy and the FDI 1mVSTIM MEP(*r* = –0.364, *P* = 0.11), SICI (*r* = 0.102, *P* = 0.66), LICI (*r* = –0.010, *P* = 0.964) or ICF (*r* = −0.079, *P* = 0.735).

**TABLE 1:** Results of the A1 block of gameplay.

	Accuracy (% correct notes played)	Best continuous streak (no. of notes played)	No. of overstrums
1st three songs (average)	51.2 ± 28.5%	29.2 ± 30.8	132.0 ± 85.7
2nd three songs (average)	61.8 ± 24.8%^*^	36.3 ± 34.9^*^	119.1 ± 86.2^*^

*Note*: The data are means ± SD. The performance measures were averaged over the first three and last three songs of the block, for comparison.

* Significantly different; *P *< 0.05.

## 4 DISCUSSION

The main aim of the present study was to determine the effects of matched duration pre‐ and post‐activity rest periods on measures of CSE. We hypothesized that the introduction of time‐matched pre‐ and post‐activity rest would support an increase in CSE immediately after activity and after post‐activity rest. The hypothesis was supported by the main finding of the study, which was that corticospinal excitability was increased in the FDI between R1 and R2 and between A1 and R2. However, to the surprise of the researchers, there was no significant difference in MEP between R1 and A1, and no significant difference in the ADM measures across any of the time periods. Further analysis of the videogame performance during A1 revealed that the subjects significantly improved gameplay performance, showing there were some short‐term performance improvements during the 30 min of activity. Though not directly correlated, the data recorded from participants showed both increases in corticospinal excitability and improvement in the task. This provides further data in regard to the effect rest can have on CSE changes as well as suggesting no reliable or detectable changes found between CSE and intracortical inhibitory/facilitatory measures in the presence of short‐term motor skill performance.

### 4.1 Motor evoked potential and short‐term motor performance

Although not statistically significant, the observed increase in FDI MEP amplitude of the active hand following motor task performance was consistent with previous studies (Cirillo et al., 2011; Kantak et al., 2013; Winkler et al., 2012). While not directly measured, both spinal and supraspinal mechanisms may have contributed to this increase in MEP amplitude. For example, modulation of polysynaptic pathways at the level of the spinal cord and brainstem through input from proprioceptive circuits, as well as changes in motoneuron recruitment and firing behaviour following repetitive motor performance, may have contributed to the observed changes in MEP amplitude (Gandevia, 2001; Semmler, 2002). Despite this observation in the FDI, similar changes were not observed in the ADM. Although the cortical regions for FDI and ADM partially overlap, Ni et al. (2006) demonstrated task‐dependent differences in the MEP facilitation between these muscles, suggesting the same functional demand of a motor task can selectively modulate the excitability changes accompanied by the cortical activation of these muscles. Therefore, a similar dissociation between the ADM and FDI may have occurred in the present study, with the motor task preferentially facilitating the CSE of the FDI. However, it is also plausible that the absence of changes in the ADM MEP amplitude may reflect a methodological limitation. Due to the substantial delay that would have been caused by implementing a secondary stimulation protocol to localize the optimal ADM hotspot, stimulation was performed from a single FDI hotspot. We cannot exclude the possibility that a more precise ADM localization would have been more sensitive at detecting task‐related changes in ADM MEP amplitude (Jin et al., 2023). However, due to the potential CSE recovery that may have occurred during a second localization protocol, and because it remains uncertain whether muscle specific localization for the ADM would have been useful for observing meaningful changes in the MEP amplitude, a separate localization protocol was not performed.

### 4.2 Intracortical facilitation

While CSE increased following the performance of the motor task, ICF remained unchanged, with no significant differences in measures of ICF between blocks. One possible explanation is that changes in ICF become more apparent in the later stages of motor skill learning, rather than following short term motor performance, particularly during motor memory stabilization and early consolidation (Ho et al., 2022; Mooney et al., 2021). Ho et al. (2022) and Mooney et al. (2021), observed decreased ICF after a long session of motor skill learning, with unaffected measures of MEPs, and proposed that a diminished ICF was recorded in response to the beginnings of the stabilization process for the motor memory of the task. They also noted that this effect was learning specific and did not occur with motor performance alone. This is consistent with previous studies that have demonstrated that CSE increases during the later stages of motor learning following repeated practice of fine motor tasks over multiple training sessions, without necessarily observing the same changes in ICF (Christiansen et al., 2020; Miyaguchi et al., 2019; Pascual‐Leone et al., 1995). These later adaptations are thought to reflect plastic changes within intracortical and corticocortical networks that enhance excitatory output from the primary motor cortex to the corticospinal tract (CST) and other descending motor pathways following skill consolidation (Koles et al., 2016). Because the present study was designed to investigate the short‐term effects of motor task performance on measures of CSE, the experimental timeline may not have been sufficient to capture the neurophysiological adaptations associated with motor learning and early memory consolidation. Therefore, the physiological changes in ICF that emerge during these later stages of learning may not yet have been detectable.

### 4.3 Intracortical inhibition

There was no significant increase in SICI or LICI immediately following the motor task, or after 30 min of complete rest. This finding is consistent with previous work demonstrating that increases in MEP amplitude can occur independently of changes in other measures of cortical excitability, including ICI and ICF (Liepert et al., 1998). One possible explanation for this is that modulation of ICI is influenced by the timing of assessment relative to motor performance and subsequent consolidation. Reductions in GABAergic inhibition during short‐term motor performance have been proposed to enhance LTP‐like processes and give rise to greater CSE (Barron, 2021). However, alterations in local inhibitory networks during a post‐training rest period may influence the maintenance of these LTP‐like effects and contribute to changes in ICI during consolidation (Lensjo et al., 2025). Consistent with this, Berghuis et al. (2015) showed a significant change in SICI after a 24‐h retention period, despite observing an immediate reduction in inhibition after motor task performance, suggesting adaptations within inhibitory circuits may become more apparent during later stages of motor memory consolidation and not after the short‐term performance of a motor task. Methodological factors associated with paired pulse TMS may have also contributed to the absence of detectable changes in ICI. Although not directly measured in the current study, Spampinato et al. (2023) demonstrated that differences in the proportion of late I‐wave recruitment in response to the conditioning and test pulse protocol following a motor task can influence measures of ICI, potentially contributing to variability across individuals and experimental paradigms. Therefore, these findings suggest the short‐term motor performance paradigm used in the present study may not have provided a long enough post‐task window to elicit detectable change in SICI or LICI, despite the observed increase in MEP amplitude.

### 4.4 Motor evoked potential and time‐matched rest

The FDI MEP amplitude increased above baseline following task execution, and over a rest period of 30 min continued to increase, suggesting that CSE was not only able to recover, but also exhibit changes consistent with short‐term activity‐dependent increases in excitability. While the aim of the study was not to assess fatigue, throughout centrally fatiguing activities, a reduction in the efficiency of descending volley generation, caused by a delay in the recycling of CNS neurotransmitters, can decrease MEP amplitude (Brasil‐Neto et al., 1994; Loscher & Nordlund, 2002). However, providing sufficient rest may give time to the system to restore corticospinal responsiveness in an effort to adapt and manage the demands placed upon it (Gandevia, 2001). Most studies have examined changes in excitability experienced immediately before and after activity but have not included measures that consider an initial rest period and longer post‐activity rest interval. Kavehee et al. (2019) investigated the contribution of supraspinal centres to central fatigue and observed that a fatiguing activity decreased MEP amplitude immediately following the task, with no recovery during the subsequent 10–20 min after the task. The longer rest period used in the present study may therefore have allowed sufficient time for both fatigue resolution and activity‐dependent neural adaptation. Although participants were distracted to minimize conscious rehearsal of the task, offline neural processes associated with early motor memory consolidation may have continued to strengthen the neural circuits engaged during the task performance (Fioravante & Regehr, 2011). While not directly measured, these offline processes may have enhanced corticospinal excitability by increasing the effectiveness and synchrony of the neural circuits engaged during task performance. However, because motor consolidation was not directly assessed, this explanation should be interpreted with caution. The initial rest period may have also influenced the size of the post‐task MEP response by reducing the impact of delayed synaptic recycling and changes in membrane conductance potentially caused by the intense activation of the neurons during the task. However, the present study did not include a comparison condition without this initial rest period, and therefore it is difficult to determine the specific contribution of this rest period (Christiansen et al., 2020; Paulus & Rothwell, 2016). Although further research is needed to distinguish the relative contributions of these mechanisms, the present findings suggest that the increase in CSE observed 30 min after task completion, compared with immediately after the task, likely reflects an interaction between recovery from central fatigue and short‐term activity dependent neuroplasticity.

### 4.5 Conclusion

As with many non‐invasive stimulation techniques, it seems that the interpretability of the results is heavily reliant on the variability of the measures performed and the model used (Kalmar, 2018). Variability can be introduced through task selection and the various components of the task such as timing, intensity and goal orientation. This variability likely contributed to the lack of associations between CSE and gameplay measures, despite both changing. Using a single visit paired with the performance of a novel task allowed for the exploration of our main aims; however, it also included its own challenges regarding the limited changes in the CSE measures that can be observed with a single bout of activity. Additionally, it would have reinforced the argument of rest periods stabilizing motor memories and ultimately enhancing skill acquisition if follow‐up or retention measures were performed on the gameplay. Future studies should focus more on the link between the behavioural outcomes and the cortical measures with prescribed rest, as opposed to using the gameplay more as a stimulus to change cortical activity as was done in the current study.

Overall, this study highlights the effects rest can have on increasing CSE, following the performance of a fine motor task. Attention in future studies should be given to the timing and duration of rest periods around motor tasks and how this may aid with defending against the onset of central fatigue, and the effects this may have on long term learning. This will be particularly important for patients with neurological diseases that require sufficient levels of excitability to attain performance‐induced learning from therapy.

## AUTHOR CONTRIBUTIONS

Quinn McCallion and Zachary A. Riley contributed to the conception or design of the work, acquisition, analysis or interpretation of data for the work, and drafting the work or revising it critically for important intellectual content. Alistair R. Clark, Bryan S. Barry, Emelia E. Duchow, and Brach Poston contributed to the acquisition, analysis or interpretation of data for the work, and drafting the work or revising it critically for important intellectual content. All authors have read and approved the final version of this manuscript and agree to be accountable for all aspects of the work in ensuring that questions related to the accuracy or integrity of any part of the work are appropriately investigated and resolved. All persons designated as authors qualify for authorship, and all those who qualify for authorship are listed.

## CONFLICT OF INTEREST

None declared.

## FUNDING INFORMATION

No funding was obtained for this work.

## GENERATIVE AI STATEMENT

No generative AI tools were used in the preparation of this manuscript

## Data Availability

The shared data has been uploaded to Harvard dataverse.
